# Low brain-derived neurotrophic factor and high vascular cell adhesion molecule-1 levels are associated with chronic kidney disease in patients with type 2 diabetes mellitus

**DOI:** 10.3389/fendo.2024.1403717

**Published:** 2024-09-17

**Authors:** Yu-Hsin Chiang, Yu-Hsuan Li, Yin-Ching Chan, Yu-Cheng Cheng, Junyi Wu, Jer-An Lin, Wei-Chang Huang, I-Te Lee

**Affiliations:** ^1^ Department of Education, Taichung Veterans General Hospital, Taichung, Taiwan; ^2^ School of Medicine, Chung Shan Medical University, Taichung, Taiwan; ^3^ Division of Endocrinology and Metabolism, Department of Internal Medicine, Taichung Veterans General Hospital, Taichung, Taiwan; ^4^ School of Medicine, National Yang Ming Chiao Tung University, Taipei, Taiwan; ^5^ Department of Computer Science & Information Engineering, National Taiwan University, Taipei, Taiwan; ^6^ Department of Food and Nutrition, Providence University, Taichung, Taiwan; ^7^ Institute of Biomedical Sciences, National Chung Hsing University, Taichung, Taiwan; ^8^ Graduate Institute of Food Safety, National Chung Hsing University, Taichung, Taiwan; ^9^ Department of Chest Medicine, Taichung Veterans General Hospital, Taichung, Taiwan; ^10^ Mycobacterial Center, Taichung Veterans General Hospital, Taichung, Taiwan; ^11^ Department of Post-Baccalaureate Medicine, College of Medicine, National Chung Hsing University, Taichung, Taiwan

**Keywords:** brain-derived neurotrophic factor, chronic kidney disease, synergistic effect, type 2 diabetes mellitus, vascular cell adhesion molecule-1

## Abstract

**Background:**

Patients with type 2 diabetes mellitus (DM) have a high prevalence of chronic kidney disease (CKD). Energy imbalance and inflammation may be involved in the pathogenesis of CKD. We examined the effects of brain-derived neurotrophic factor (BDNF) and vascular cell adhesion molecule-1 (VCAM-1) on CKD in patients with type 2 DM.

**Methods:**

Patients with type 2 DM were enrolled for this cross-sectional study. Fasting serum was prepared to measure the BDNF and VCAM-1 levels. An estimated glomerular filtration rate (eGFR) <60 mL/min/1.73 m^2^ was used as the criterion for identifying patients with CKD.

**Results:**

Of the 548 enrolled participants, 156 had CKD. Patients with CKD exhibited significantly lower BDNF (median of 21.4 ng/mL, interquartile range [IQR]: 17.0–27.0 ng/mL vs. median of 25.9 ng/mL, IQR: 21.0–30.4 ng/mL, *P <*0.001) and higher VCAM-1 (median of 917 ng/mL, IQR: 761–1172 ng/mL vs. median of 669 ng/mL, IQR: 552–857 ng/mL, *P <*0.001) levels than those without CKD. Serum BDNF levels were inversely correlated with VCAM-1 levels (Spearman’s rank correlation coefficient = -0.210, *P <*0.001). The patients were divided into four subgroups based on median BDNF and VCAM-1 levels (24.88 ng/mL and 750 ng/mL, respectively). Notably, patients in the high VCAM-1 and low BDNF group had the highest prevalence (50%) of CKD. Multivariate logistic regression revealed a significantly higher odds ratio (OR) of CKD in the high VCAM-1 and low BDNF group (OR = 3.885, 95% CI: 1.766–8.547, *P <*0.001), followed by that in the high VCAM-1 and high BDNF group (OR = 3.099, 95% CI: 1.373–6.992, *P* =0.006) compared with that in the low VCAM-1 and high BDNF group. However, the risk of CKD in the low VCAM-1 and low BDNF group was not significantly different from that in the low VCAM-1 and high BDNF group (*P* =0.266).

**Conclusion:**

CKD in patients with type 2 DM is associated with low serum BDNF and high VCAM-1 levels. BDNF and VCAM-1 have a synergistic effect on CKD. Thus, BDNF and VCAM-1 can be potential biomarkers for CKD risk stratification in patients with type 2 DM.

## 1 Introduction

Chronic kidney disease (CKD) poses an increasing public health concern. The global prevalence of patients with an estimated glomerular filtration rate (eGFR) <60 mL/min/1.73 m^2^ was approximately 4% in 2017 (1). The high mortality rate and costs associated with CKD, coupled with increasing dependency on caregivers, become considerable challenge (2–4). According to the United States Renal Data System annual data report for 2021, 59.9% of the patients with incident end-stage kidney disease had diabetes mellitus (DM) (5). According to the data from the National Health and Nutrition Examination Survey 2007–2014 (6), approximately 18% of patients with type 2 DM had an eGFR <60 mL/min/1.73 m^2^. Therefore, there is a notable correlation between CKD and type 2 DM.

Brain-derived neurotrophic factor (BDNF) plays a crucial role in energy homeostasis in humans. Circulating BDNF levels are significantly lower in patients with type 2 DM than in healthy controls (7, 8). Moreover, low serum BDNF levels have been linked to various chronic complications of DM, including diabetic retinopathy and neuropathy (9, 10). Interestingly, a notable correlation has been found between eGFR and serum BDNF levels in individuals without DM (11). However, the association between BDNF and CKD in patients with type 2 DM remains unexplored.

Vascular cell adhesion molecule-1 (VCAM-1) is a glycoprotein expressed in endothelial cells (12) and promotes the attachment of white blood cells to the inner lining of blood vessels (13). It has been reported that serum VCAM-1 levels are notably increased in patients with type 2 DM (14). Moreover, this increase was associated with the development of diabetic microvascular complications (14–16).

In patients without known DM, low serum BDNF levels have been reported to be associated with high VCAM-1 levels (17). However, patients with type 2 DM have significantly lower BDNF and higher VCAM-1 levels than healthy controls (8, 14). We hypothesized that circulating BDNF levels are inversely associated with VCAM-1 in patients with type 2 DM, and that this association plays a role in CKD. Therefore, we conducted a cross-sectional study on patients with type 2 DM and examined the risk of CKD categorized according to serum BDNF and VCAM-1 levels.

## 2 Materials and methods

### 2.1 Patients

This cross-sectional study was conducted at Taichung Veterans General Hospital. Patients were enrolled from the outpatient department between March 2018 and October 2018. The inclusion criteria were as follows: age ≥45 years, clinical diagnosis of DM by physicians, and poor glucose control (defined as at least one incidence of glycated hemoglobin [HbA1c] levels >9.0% within previous one year based on the medical record). Patients with the following status at enrollment were excluded: diabetic classification other than type 2 DM, C-reactive protein (CRP) levels >60 mg/L, chronic active infection such as tuberculosis, history of immunocompromise or use of immunosuppressors, and incomplete study assessments. After reviewing the medical history and anthropometric measurements, blood and spot urine samples were collected following overnight fasting. This study was approved by the Institutional Review Board of Taichung Veterans General Hospital, and written informed consent was obtained from all participants.

### 2.2 Biochemical assessment

Plasma glucose levels were determined using the glucose oxidase-peroxidase method (Wako Diagnostics, Tokyo, Japan). HbA1c levels were determined using boronate-affinity high-performance liquid chromatography (NGSP-certified, Primus Corp., Kansas City, MO, USA). Serum creatinine, CRP, and lipid levels were determined using commercial kits (Beckman Coulter, Fullerton, CA, USA). Serum free BDNF levels were determined using an immunoassay kit (R&D Systems, Minneapolis, MN, USA), with an intra-assay coefficient of variation (CV) of 6.2% and an inter-assay CV of 8.1%. Serum VCAM-1 levels were determined using an immunoassay kit (R&D Systems, Minneapolis, MN, USA), with an intra-assay CV of 2.3% and an inter-assay CV of 7.8%. Urinary albumin levels were evaluated using the polyethylene glycol-enhanced immunoturbidimetric method (Advia 1800 system, Siemens, NY, USA).

The eGFR was calculated using the Chronic Kidney Disease Epidemiology Collaboration equation (18). CKD was defined as a condition with an eGFR <60 mL/min/1.73 m^2^. The urine albumin-to-creatinine ratio (UACR) was calculated by dividing the levels of urine albumin (mg) with those of urine creatinine (g). Increased albuminuria was defined as a UACR ≥30 mg/g (19). Obesity was defined as a body mass index (BMI) ≥27 kg/m^2^ (20). Hypertension was characterized by systolic blood pressure ≥130 mmHg, diastolic blood pressure ≥80 mmHg, or a documented history of antihypertensive medication use. Low high-density lipoprotein (HDL) cholesterol was defined as an HDL level <1.0 mmol/L (40 mg/dL) in men and <1.3 mmol/L (50 mg/dL) in women. Hypercholesterolemia was defined as a total cholesterol level ≥4.14 mmol/L (160 mg/dL) or a low-density lipoprotein (LDL) cholesterol level ≥2.59 mmol/L (100 mg/dL). Hypertriglyceridemia was defined as a triglyceride level ≥1.7 mmol/L (150 mg/dL) (21).

### 2.3 Statistical analyses

Continuous variables are presented as means along with the corresponding standard deviations, whereas data of UACR, BDNF, and VCAM-1 in patients with or without CKD are presented as medians along with interquartile range (IQR). Categorical variables are presented as numbers along with their respective percentages. Because the assessed continuous variables did not exhibit normal distribution based on the Kolmogorov-Smirnov test, we employed the Mann-Whitney U test to assess the differences in continuous variables between individuals with and without CKD. Simultaneously, a Mann-Whitney U test was applied to examine the differences in serum BDNF and VCAM-1 levels between the paired groups categorized according to the risk factors associated with CKD. Spearman’s rank correlation coefficient (rho) was used to determine the correlation between continuous variables. A trend test was used to examine the changes in the ratio of serum BDNF to VCAM-1 across the groups categorized by eGFR. We compared the CKD risks in different models by examining the C-index. The performance of the index in BDNF + VCAM-1 model was compared with that of the BDNF or VCAM-1 alone model. Furthermore, the patients were divided into four subgroups based on median BDNF and VCAM-1 levels. A trend test was performed to examine the linearly increasing trend in CKD prevalence among the four subgroups. In addition, univariate logistic regression analyses were used to explore the odds ratios (OR) and 95% confidence interval (CI) of CKD for the four subgroups and the quartiles of BDNF or VCAM-1. In the multivariate regression analysis, the assessed variables which were significantly associated with both CKD and the BDNF or VCAM-1 levels were included. Statistical analyses were performed using the SPSS version 22.0 software (IBM Corp., Armonk, NY, USA), and C index was assessed using R software v3.4.

## 3 Results

Among the 548 participants enrolled in this study, 156 had CKD (CKD group), and 392 did not have CKD (non-CKD group). **Table 1** presents the baseline characteristics of the patients in these two groups. Patients with CKD had significantly lower serum BDNF (median of 21.4 ng/mL, IQR: 17.0–27.0 ng/mL vs. median of 25.9 ng/mL, IQR: 21.0–30.4 ng/mL, *P* < 0.001) and higher serum VCAM-1 (median of 917 ng/mL, IQR: 761–1172 ng/mL vs. median of 669 ng/mL, IQR: 552–857 ng/mL, *P* < 0.001) levels than those without CKD. Patients with CKD were significantly older (69.7 ± 9.0 vs. 60.4 ± 8.5 years, *P* < 0.001) and mostly males (66.0% vs. 53.3%, *P* = 0.009). Patients with CKD had significantly lower diastolic blood pressure (71 ± 13 vs. 75 ± 11 mmHg, *P* < 0.001), lower total cholesterol (3.9 ± 0.9 vs. 4.2 ± 1.0 mmol/L, *P* < 0.001), lower LDL cholesterol (2.1 ± 0.7 vs. 2.4 ± 0.8 mmol/L, *P* < 0.001), and lower HDL cholesterol (1.2 ± 0.3 vs. 1.3 ± 0.4 mmol/L, *P* < 0.001) levels, but higher triglyceride levels (1.9 ± 1.5 vs. 1.6 ± 1.2 mmol/L, *P* < 0.001). In addition to a significantly lower eGFR (48.0 ± 11.2 vs. 78.7 ± 11.6 mL/min/1.73 m^2^, *P* < 0.001), the CKD group had a significantly higher UACR (median of 132 mg/g, IQR: 24–448 mg/g) in comparison with that (median of 19 mg/g, IQR: 8–74 mg/g; *P* < 0.001) in the non-CKD group. Patients with CKD had significantly lower levels of alanine aminotransferase (24 ± 16 vs. 28 ± 23 U/L, *P* = 0.006) than those without CKD. Regarding the current use of medications, the CKD group had significantly higher proportions of patients using antiplatelet drugs (20.5% vs. 11.7%, *P* = 0.012), antihypertensive drugs (53.8% vs. 34.2%, *P* < 0.001), and insulin injection (55.8% vs. 45.4%, *P* = 0.036). The proportion of patients using oral antihyperglycemic drugs was not significantly different (*P* = 0.626) between groups; however, the CKD group had significantly lower proportions of patients using metformin (*P* < 0.001) and sodium-glucose cotransporter 2 inhibitors (*P* = 0.002) but a significantly higher proportion of patients using dipeptidyl peptidase-4 inhibitors (*P* = 0.036) than the non-CKD group. BMI, systolic blood pressure, glucose, HbA1c, and CRP levels, proportion of current smokers, and proportions of patients with previous coronary artery disease (CAD) history, hypertension, use of statins, and use of glucagon-like peptide-1 receptor agonists were not significantly different (*P* > 0.05) between the CKD and non-CKD groups.

**Table 1:** Characteristics of enrolled patients with and without CKD.

	CKD (n = 156)	non-CKD (n = 392)	*P*
Age (year)	69.7 ± 9.0	60.4 ± 8.5	<0.001
Male, *n* (%)	103 ± (66.0%)	209 ± (53.3%)	0.009
Current smoking, *n* (%)	11 ± (7.1%)	46 ± (11.7%)	0.143
CAD, *n* (%)	21 ± (13.5%)	30 ± (7.7%)	0.251
BMI (kg/m^2^)	26.5 ± 4.3	25.9 ± 4.4	0.144
Systolic BP (mmHg)	134 ± 21	132 ± 16	0.150
Diastolic BP (mmHg)	71 ± 13	75 ± 11	<0.001
Fasting glucose (mmol/L)	8.3 ± 2.7	8.9 ± 3.2	0.053
HbA1c (%)	8.5 ± 1.6	8.7 ± 1.6	0.203
Total cholesterol (mmol/L)	3.9 ± 0.9	4.2 ± 1.0	<0.001
LDL cholesterol (mmol/L)	2.1 ± 0.7	2.4 ± 0.8	<0.001
HDL cholesterol (mmol/L)	1.2 ± 0.3	1.3 ± 0.4	<0.001
Triglyceride (mmol/L)	1.9 ± 1.5	1.6 ± 1.2	<0.001
eGFR (mL/min/1.73 m^2^)	48.0 ± 11.2	78.7 ± 11.6	<0.001
UACR (mg/g)*	132 ± (24–448)	19 ± (8–74)	<0.001
Alanine aminotransferase (U/L)	24 ± 16	28 ± 23	0.006
C-reactive protein; (mg/L)	3.3 ± 6.4	2.0 ± 3.0	0.190
BDNF (ng/mL)*	21.4 ± (17.0–27.0)	25.9 ± (21.0–30.4)	<0.001
VCAM-1 (ng/mL)*	917 ± (761–1172)	669 ± (552–857)	<0.001
Use of antiplatelet agents, *n* (%)	32 ± (20.5%)	46 ± (11.7%)	0.012
Use of statins, *n* (%)	86 ± (55.1%)	246 ± (62.8%)	0.121
Hypertension, *n* (%)	128 ± (82.1%)	290 ± (74.0%)	0.058
Use of antihypertensive agents, *n* (%)	84 ± (53.8%)	134 ± (34.2%)	<0.001
ACE inhibitor or ARB, *n* (%)	68 ± (43.6%)	118 ± (30.1%)	0.004
α-blocker, *n* (%)	7 ± (4.5%)	10 ± (2.6%)	0.365
β-blocker, *n* (%)	18 ± (11.5%)	16 ± (4.1%)	0.002
Calcium channel blocker, *n* (%)	41 ± (26.3%)	45 ± (11.5%)	<0.001
Diuretics, *n* (%)	25 ± (16.0%)	17 ± (4.3%)	<0.001
Use of insulin therapy, *n* (%)	87 ± (55.8%)	178 ± (45.4%)	0.036
Use of GLP-1 RA, *n* (%)	15 ± (9.6%)	25 ± (6.4%)	0.257
Use of oral antihyperglycemic drugs, *n* (%)	138 ± (88.5%)	354 ± (90.3%)	0.626
Insulin secretagogues, *n* (%)	83 ± (53.2%)	185 ± (47.2%)	0.240
Metformin, *n* (%)	79 ± (50.6%)	292 ± (74.5%)	<0.001
Thiazolidinediones, *n* (%)	40 ± (25.6%)	89 ± (22.7%)	0.535
α-Glucosidase inhibitor, *n* (%)	8 ± (5.1%)	18 ± (4.6%)	0.965
DPP4 inhibitors, *n* (%)	91 ± (58.3%)	188 ± (48.0%)	0.036
SGLT2 inhibitors, *n* (%)	11 ± (7.1%)	71 ± (18.1%)	0.002

ACE, angiotensin-converting enzyme; ARB, angiotensin II receptor blocker; BDNF, brain-derived neurotrophic factor; BMI, body mass index; BP, blood pressure; CAD, coronary artery disease; CKD, chronic kidney disease; DPP4, dipeptidyl peptidase-4; eGFR, estimated glomerular filtration rate; GLP-1 RA, glucagon-like peptide-1 receptor agonist; HbA1c, glycated hemoglobin; HDL, high-density lipoprotein; LDL, low-density lipoprotein; SGLT2, sodium-glucose cotransporter 2; UACR, urinary albumin-to-creatinine ratio; VCAM-1, vascular cell adhesion molecule-1.

*UACR, BDNF, and VCAM-1 are presented as median (interquartile range) due to skewed distributions.

**Figure 1:**
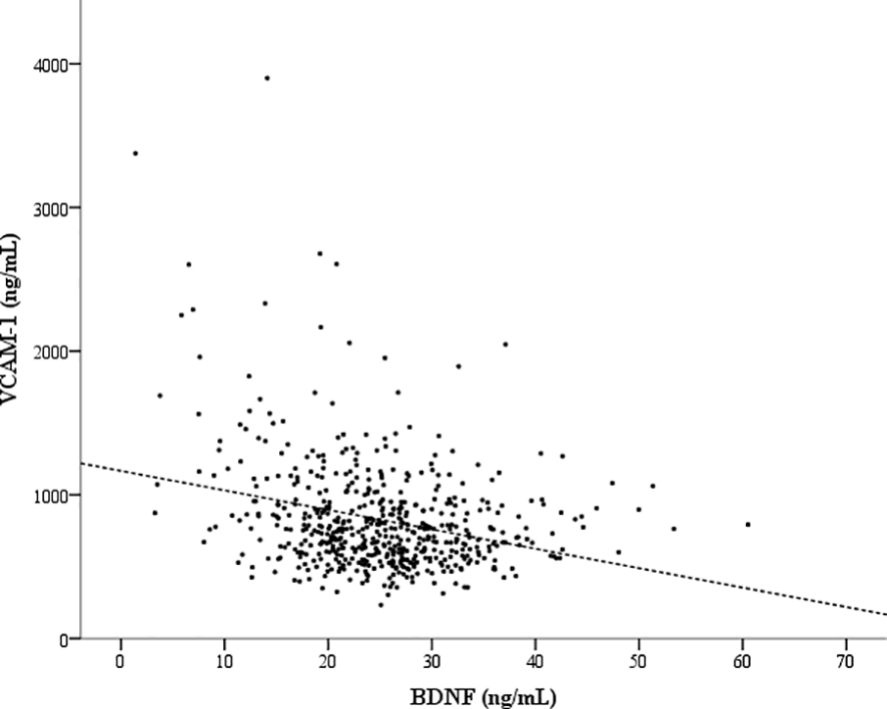
An inverse correlation between the serum brain-derived neurotrophic factor (BDNF) and vascular cell adhesion molecule-1 (VCAM-1) levels. Spearman’s rank correlation coefficient (rho) was -0.210 (*P* < 0.001) for the correlation between the serum BDNF and VCAM-1 levels.

To highlight the potential factors that might be associated with BDNF or VCAM-1, we have listed the serum BDNF or VCAM-1 levels in patients categorized by the assessed factors in **Table 2**. Young age, female sex, hyperglycemia (including fasting glucose and HbA1c), high total cholesterol, hypertriglyceridemia, normal UACR, use of statins and metformin, and no use of α-glucosidase inhibitor were significantly associated with high serum BDNF levels. Moreover, age, CAD history, hypertension, low cholesterol (including total, LDL, and HDL cholesterol) levels, increased UACR, use of insulin therapy and antihypertensive drugs, and no use of statins or metformin were significantly associated with high serum VCAM-1 levels. We divided the patients into two groups based on median serum BDNF level (<24.88 ng/mL and ≥24.88 ng/mL), and the group with lower BDNF levels exhibited higher serum VCAM-1 levels (906 ± 463 vs. 750 ± 272 ng/mL, *P <*0.001) compared with the group with higher BDNF levels. Similarly, we divided the patients into two groups based on median serum VCAM-1 levels (<750 ng/mL and ≥750 ng/mL), and the group with lower VCAM-1 levels displayed higher serum BDNF levels (26.2 ± 6.5 vs. 23.8 ± 9.1 ng/mL, *P* < 0.001) than that in the group with higher VCAM-1 levels. Serum VCAM-1 levels showed a significant inverse correlation with serum BDNF levels (rho = -0.210, *P* < 0.001; **Figure 1**).

**Table 2:** Serum BDNF and VCAM-1 levels in patients grouped based on CKD risk factors.

	Group	Patient number	BDNF (ng/mL)	*P*	VCAM-1 (ng/mL)	*P*
Age	< 65 years	(*n* = 320)	26.6 ± 7.6	<0.001	734 ± 292	<0.001
	≥ 65 years	(*n* = 228)	22.7 ± 8.0		959 ± 461	
Sex	Female	(*n* = 236)	26.2 ± 7.8	0.003	798 ± 335	0.340
	Male	(*n* = 312)	24.1 ± 8.1		850 ± 421	
Current smokers	No	(*n* = 491)	25.0 ± 8.0	0.891	830 ± 390	0.405
	Yes	(*n* = 57)	24.8 ± 7.6		809 ± 364	
CAD	No	(*n* = 497)	25.0 ± 7.9	0.877	816 ± 387	0.007
	Yes	(*n* = 51)	25.1 ± 9.0		940 ± 376	
Hypertension	No	(*n* = 130)	25.1 ± 8.0	0.656	774 ± 342	0.013
	Yes	(*n* = 418)	25.0 ± 8.0		845 ± 399	
BMI	< 27 kg/m^2^	(*n* = 341)	24.5 ± 8.1	0.144	851 ± 416	0.147
	≥ 27 kg/m^2^	(*n* = 207)	25.8 ± 7.7		789 ± 332	
Systolic BP	< 130 mmHg	(*n* = 237)	24.8 ± 7.9	0.771	805 ± 394	0.052
	≥ 130 mmHg	(*n* = 311)	25.1 ± 8.1		845 ± 382	
Diastolic BP	< 80 mmHg	(*n* = 373)	24.5 ± 8.0	0.105	852 ± 421	0.133
	≥ 80 mmHg	(*n* = 175)	26.0 ± 7.8		775 ± 296	
Fasting glucose	< 7.2 mmol/L	(*n* = 188)	23.2 ± 7.6	<0.001	828 ± 372	0.925
	≥ 7.2 mmol/L	(*n* = 360)	25.9 ± 8.0		827 ± 396	
HbA1c	< 8.5%	(*n* = 282)	23.7 ± 7.6	<0.001	820 ± 403	0.114
	≥ 8.5%	(*n* = 266)	26.4 ± 8.2		836 ± 370	
Total cholesterol	< 4.14 mmol/L	(*n* = 306)	24.0 ± 7.8	<0.001	882 ± 436	<0.001
	≥ 4.14 mmol/L	(*n* = 242)	26.3 ± 8.1		758 ± 302	
LDL cholesterol	< 2.59 mmol/L	(*n* = 382)	24.5 ± 7.8	0.054	871 ± 429	<0.001
	≥ 2.59 mmol/L	(*n* = 166)	26.1 ± 8.3		727 ± 241	
Low HDL cholesterol*	No	(*n* = 353)	24.6 ± 8.2	0.381	799 ± 387	<0.001
	Yes	(*n* = 195)	25.6 ± 7.6		880 ± 382	
Triglycerides	< 1.7 mmol/L	(*n* = 346)	24.2 ± 7.9	0.007	839 ± 430	0.728
	≥ 1.7 mmol/L	(*n* = 202)	26.4 ± 8.0		808 ± 300	
Alanine aminotransferase	< 20 U/L	(*n* = 226)	24.9 ± 7.3	0.936	801 ± 292	0.892
	≥ 20 U/L	(*n* = 322)	25.0 ± 8.5		846 ± 442	
BDNF	< 24.88 ng/mL	(*n* = 274)	18.9 ± 4.7	<0.001	906 ± 463	<0.001
	≥ 24.88 ng/mL	(*n* = 274)	31.1 ± 5.6		750 ± 272	
VCAM-1	< 750 ng/mL	(*n* = 274)	26.2 ± 6.5	<0.001	578 ± 101	<0.001
	≥ 750 ng/mL	(*n* = 274)	23.8 ± 9.1		1077 ± 406	
C-reactive protein;	< 1.11 mg/L	(*n* = 273)	25.1 ± 7.7	0.835	810 ± 386	0.124
	≥ 1.11 mg/L	(*n* = 275)	24.9 ± 8.3		846 ± 388	
UACR	< 30 mg/g	(*n* = 282)	25.7 ± 7.6	0.019	727 ± 257	<0.001
	≥ 30 mg/g	(*n* = 266)	24.2 ± 8.3		934 ± 466	
Use of statins	No	(*n* = 216)	23.6 ± 8.3	0.001	891 ± 438	0.006
	Yes	(*n* = 332)	25.9 ± 7.6		786 ± 345	
Use of antiplatelet drugs	No	(*n* = 470)	25.0 ± 7.9	0.610	822 ± 397	0.073
	Yes	(*n* = 78)	24.7 ± 8.6		862 ± 321	
Use of antihypertensive drugs	No	(*n* = 330)	25.1 ± 8.2	0.458	796 ± 370	<0.001
	Yes	(*n* = 218)	24.8 ± 7.6		876 ± 408	
ACE inhibitor or ARB	No	(*n* = 362)	25.1 ± 8.2	0.590	803 ± 368	0.003
	Yes	(*n* = 186)	24.9 ± 7.6		876 ± 419	
α-blocker	No	(*n* = 531)	25.0 ± 8.0	0.522	826 ± 390	0.282
	Yes	(*n* = 17)	23.9 ± 6.9		871 ± 287	
β-blocker	No	(*n* = 514)	25.1 ± 8.1	0.428	824 ± 393	0.074
	Yes	(*n* = 34)	24.2 ± 6.1		878 ± 284	
Calcium channel blocker	No	(*n* = 462)	25.1 ± 8.0	0.328	822 ± 402	0.037
	Yes	(*n* = 86)	24.5 ± 7.9		858 ± 295	
Diuretics	No	(*n* = 506)	24.9 ± 8.0	0.336	821 ± 393	0.010
	Yes	(*n* = 42)	26.3 ± 7.6		908 ± 299	
Use of insulin therapy	No	(*n* = 283)	24.6 ± 7.6	0.347	770 ± 332	<0.001
	Yes	(*n* = 265)	25.4 ± 8.4		889 ± 431	
Use of GLP-1 agonists	No	(*n* = 508)	25.0 ± 8.0	0.926	836 ± 395	0.053
	Yes	(*n* = 40)	25.3 ± 8.3		719 ± 242	
Use of oral antidiabetic drugs	No	(*n* = 56)	26.2 ± 7.8	0.351	832 ± 314	0.427
	Yes	(*n* = 492)	24.9 ± 8.0		827 ± 395	
Use of insulin secretagogues	No	(*n* = 280)	25.1 ± 7.3	0.784	828 ± 365	0.997
	Yes	(*n* = 268)	24.9 ± 8.7		827 ± 410	
Metformin	No	(*n* = 177)	24.2 ± 8.4	0.027	927 ± 456	<0.001
	Yes	(*n* = 371)	25.4 ± 7.8		780 ± 340	
Thiazolidinediones	No	(*n* = 419)	25.2 ± 8.1	0.230	830 ± 393	0.810
	Yes	(*n* = 129)	24.2 ± 7.7		820 ± 369	
α-Glucosidase inhibitor	No	(*n* = 522)	25.2 ± 8.1	0.034	826 ± 392	0.257
	Yes	(*n* = 26)	21.9 ± 5.8		853 ± 270	
DPP4 inhibitors	No	(*n* = 269)	25.4 ± 8.0	0.217	809 ± 379	0.221
	Yes	(*n* = 279)	24.6 ± 8.0		846 ± 395	
SGLT2 inhibitors	No	(*n* = 466)	24.8 ± 8.1	0.201	839 ± 403	0.182
	Yes	(*n* = 82)	25.9 ± 7.4		765 ± 279	

ACE, angiotensin-converting enzyme; ARB, angiotensin II receptor blocker; BDNF, brain-derived neurotrophic factor; BMI, body mass index; BP, blood pressure; CAD, coronary artery disease; CKD, chronic kidney disease; DPP4, dipeptidyl peptidase-4; eGFR, estimated glomerular filtration rate; GLP-1 RA, glucagon-like peptide-1 receptor agonist; HbA1c, glycated hemoglobin; HDL, high-density lipoprotein; LDL, low-density lipoprotein; SGLT2, sodium-glucose cotransporter 2; UACR, urinary albumin-to-creatinine ratio; VCAM-1, vascular cell adhesion molecule-1.

* Low HDL cholesterol means an HDL level <1.0 mmol/L (40 mg/dL) in men or <1.3 mmol/L (50 mg/dL) in women.

To further assess the relationship of CKD-associated characteristics with BDNF or VCAM-1, **Table 3** shows the rho values for the correlation of the continuous variables that were significantly different between the CKD and non-CKD groups (as shown in **Table 1**) with serum BDNF or VCAM-1 levels. In the CKD group, BDNF showed a significant positive correlation with eGFR (rho = 0.180, *P* = 0.024) and an inverse correlation with age (rho = -0.183, *P* = 0.022). In the non-CKD group, BDNF showed a significant positive correlation with triglycerides (rho = 0.175, *P* < 0.001), total cholesterol (rho = 0.164, *P* = 0.001), and LDL cholesterol (rho = 0.104, *P* = 0.040) and an inverse correlation with age (rho = -0.186, *P* < 0.001) and VCAM-1 (rho = -0.123, *P* = 0.014). In the CKD group, VCAM-1 had significantly positive correlation with UACR (rho = 0.248, *P* = 0.002) and age (rho = 0.233, *P* = 0.003) and an inverse correlation with eGFR (rho = -0.389, *P* < 0.001) and diastolic blood pressure (rho = -0.184, *P* = 0.021). In the non-CKD group, VCAM-1 showed a significant positive correlation with age (rho = 0.205, *P* < 0.001), UACR (rho = 0.141, *P* = 0.005), and alanine aminotransferase (rho = 0.116, *P* = 0.021) and an inverse correlation with eGFR (rho = -0.192, *P* < 0.001), total cholesterol (rho = -0.139, *P* = 0.006), and LDL cholesterol (rho = -0.137, *P* = 0.007).

**Table 3:** Spearman’s rank correlation coefficient (rho) between the CKD-associated characteristics and serum BDNF or VCAM-1 levels in the CKD and non-CKD groups.

	BDNF					VCAM-1			
	CKD (n= 156)		Non-CKD (n = 392)			CKD (n= 156)		Non-CKD (n = 392)	
VCAM-1	-0.142	(*P* = 0.078)	-0.123	(*P* = 0.014)	BDNF	-0.142	(*P* = 0.078)	-0.123	(*P* = 0.014)
Age	-0.183	(*P* = 0.022)	-0.186	(*P* < 0.001)	Age	0.233	(*P* = 0.003)	0.205	(*P* < 0.001)
Diastolic BP	0.076	(*P* = 0.347)	0.043	(*P* = 0.400)	Diastolic BP	-0.184	(*P* = 0.021)	-0.042	(*P* = 0.402)
Total cholesterol	0.113	(*P* = 0.159)	0.164	(*P* = 0.001)	Total cholesterol	-0.142	(*P* = 0.077)	-0.139	(*P* = 0.006)
LDL cholesterol	0.137	(*P* = 0.089)	0.104	(*P* = 0.040)	LDL cholesterol	-0.049	(*P* = 0.544)	-0.137	(*P* = 0.007)
HDL cholesterol	-0.054	(*P* = 0.505)	0.027	(*P* = 0.588)	HDL cholesterol	-0.037	(*P* = 0.650)	-0.089	(*P* = 0.078)
Triglycerides	0.084	(*P* = 0.296)	0.175	(*P* < 0.001)	Triglycerides	-0.068	(*P* = 0.397)	-0.040	(*P* = 0.426)
eGFR	0.180	(*P* = 0.024)	0.091	(*P* = 0.072)	eGFR	-0.389	(*P* < 0.001)	-0.192	(*P* < 0.001)
UACR	0.100	(*P* = 0.214)	-0.036	(*P* = 0.472)	UACR	0.248	(*P* = 0.002)	0.141	(*P* = 0.005)
Alanine aminotransferase	-0.047	(*P* = 0.559)	0.020	(*P* = 0.693)	Alanine aminotransferase	-0.004	(*P* = 0.956)	0.116	(*P* = 0.021)

BDNF, brain-derived neurotrophic factor; BP, blood pressure; CKD, chronic kidney disease; eGFR, estimated glomerular filtration rate; HDL, high-density lipoprotein; LDL, low-density lipoprotein; UACR, urinary albumin-to-creatinine ratio; VCAM-1, vascular cell adhesion molecule-1.

**Figure 2:**
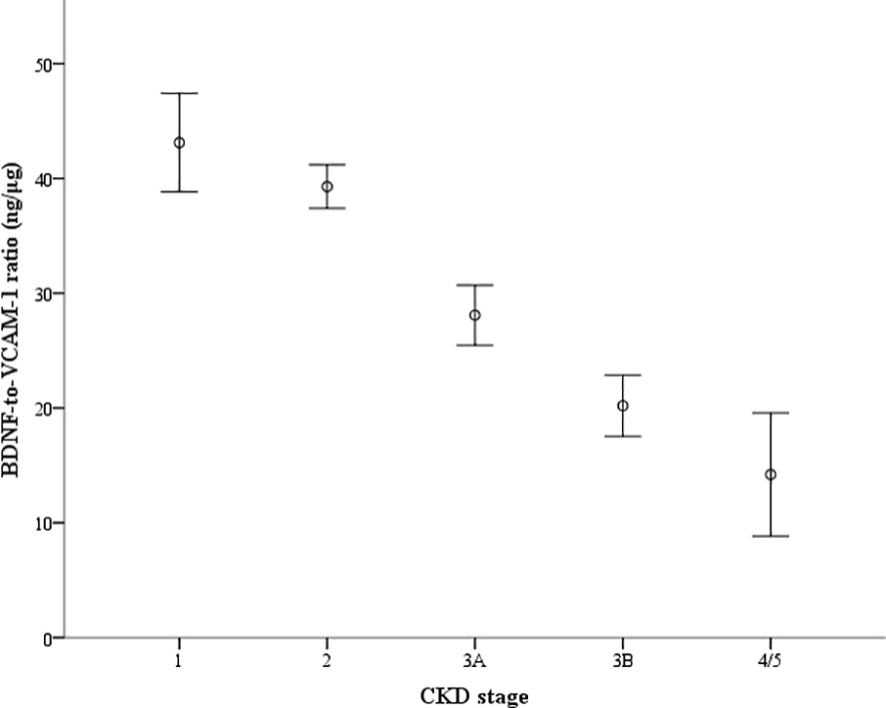
The ratio of serum brain-derived neurotrophic factor (BDNF) to vascular cell adhesion molecule-1 (VCAM-1) levels according to the groups categorized by estimated glomerular filtration rate (eGFR). The ratio (ng/μg) is depicted with the error bar showing the 95% confidence interval. The groups are staged according to eGFR as follows: stage 1 (n=65): eGFR ≥ 90 mL/min/1.73 m^2^, stage 2 (n = 327): eGFR between 60 and 89 mL/min/1.73 m^2^, stage 3A (n = 111): eGFR between 45 and 59 mL/min/1.73 m^2^, stage 3B (n = 36): eGFR between 30 and 44 mL/min/1.73 m^2^, and stage 4/5 (n=9): eGFR < 30 mL/min/1.73 m^2^). *P* value for the trend <0.001.

**Figure 3:**
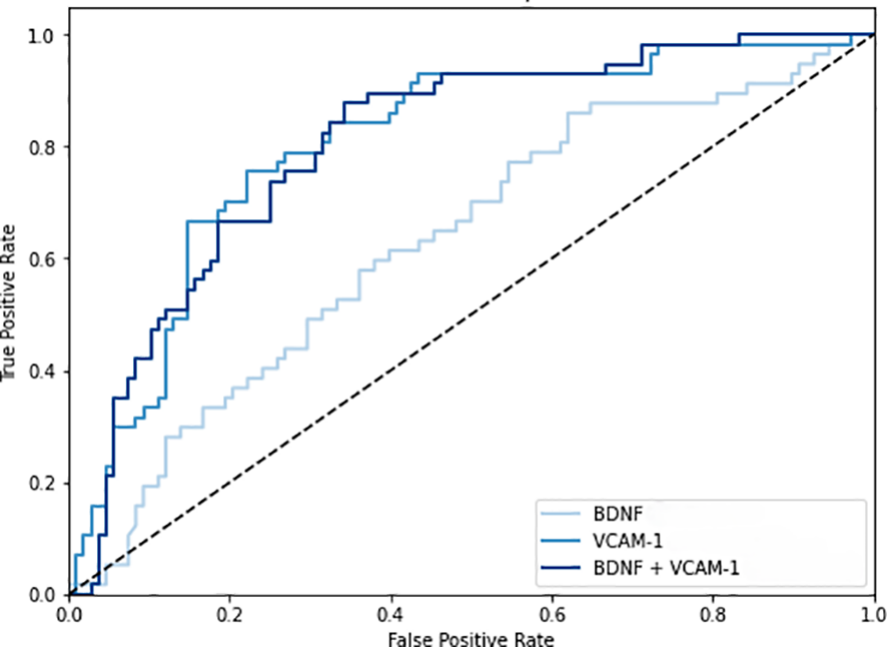
The receiver operating characteristic curves to differentiate chronic kidney disease (CKD). The curves are shown in three different models: the brain-derived neurotrophic factor (BDNF) alone model, the vascular cell adhesion molecule-1 (VCAM-1) alone model, and the BDNF + VCAM-1 model.

**Figure 2** shows the ratios of serum BDNF (ng) to VCAM-1 (μg) in patients according to their eGFR values: 43.1 ± 17.3 ng/μg in 65 patients with eGFR ≥ 90 mL/min/1.73 m^2^, 39.3 ± 17.5 ng/μg in 327 patients with eGFR between 60 and 89 mL/min/1.73m^2^, 28.1 ± 13.9 ng/μg in 111 patients with eGFR between 45 and 59 mL/min/1.73 m^2^, 20.2 ± 7.9 ng/μg in 36 patients with eGFR between 30 and 44 mL/min/1.73 m^2^, and 14.2 ± 7.0 ng/μg in 9 patients with eGFR < 30 mL/min/1.73 m^2^. The ratio of serum BDNF to VCAM-1 levels decreased as the CKD stages progressed (*P* value for the trend < 0.001). **Figure 3** shows the receiver operating characteristic curves to differentiate CKD using the BDNF alone model, the VCAM-1 alone model, and the BDNF + VCAM-1 model. The C index was 0.645 (95% CI: 0.531–0.762), 0.746 (95% CI: 0.643–0.844), and 0.756 (95% CI: 0.657–0.853); respectively. The C index is significantly higher in the BDNF + VCAM-1 model than in the BDNF alone model (*P* < 0.001), but not significantly different from that in the VCAM-1 model (*P* = 0.263).

**Figure 4:**
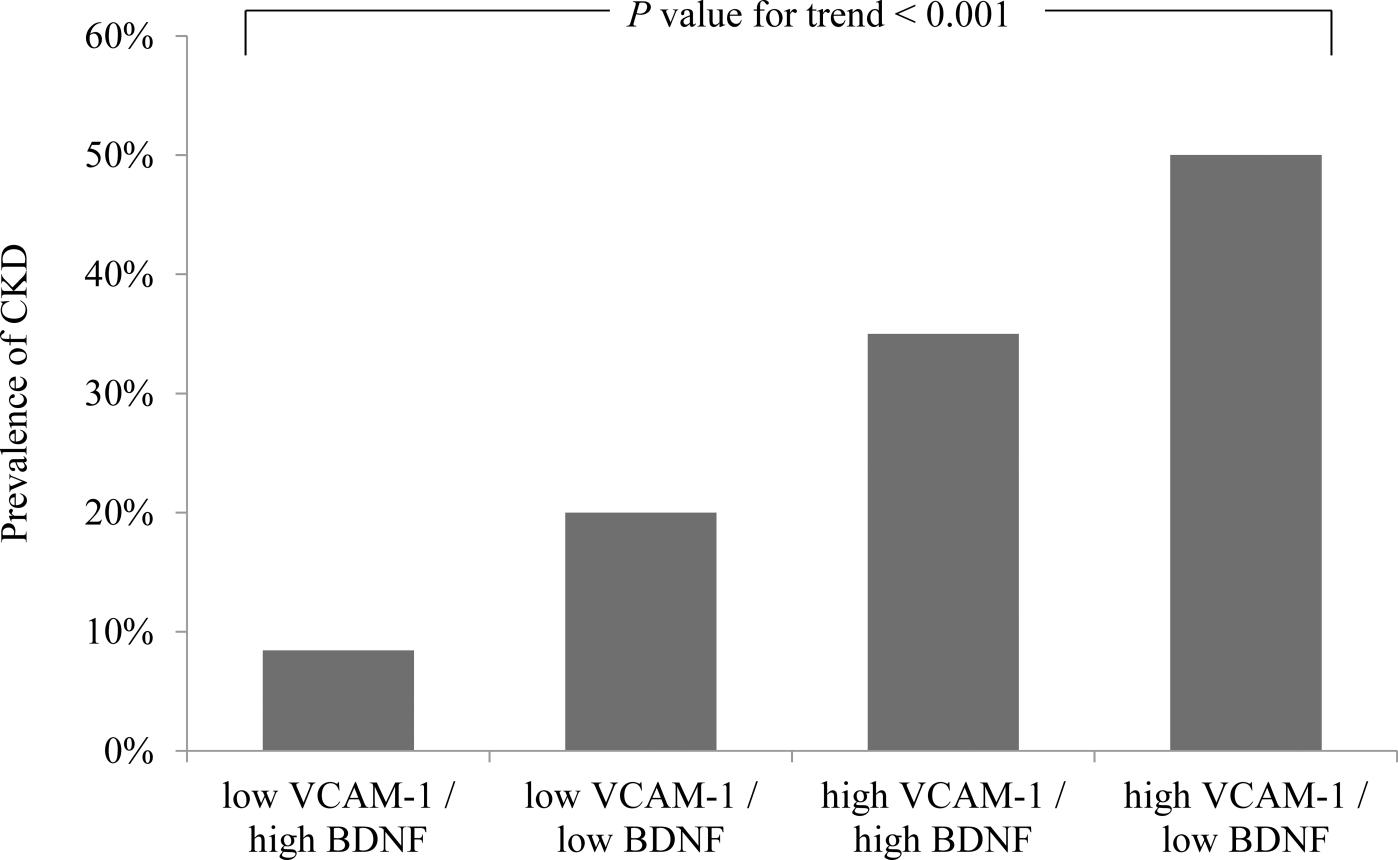
Prevalence of chronic kidney disease (CKD) according to serum brain-derived neurotrophic factor (BDNF) and vascular cell adhesion molecule-1 (VCAM-1) levels. The prevalence of CKD is shown in the four subgroups categorized by the median values of BDNF (24.88 ng/mL) and VCAM-1 (750 ng/mL).

We further divided the patients into four subgroups based on median BDNF and VCAM-1 levels. The highest prevalence of CKD (50%; 77 of 154 patients) was observed in the high VCAM-1 and low BDNF group; the prevalence of CKD was 35% (42 of 120 patients) in the high VCAM-1 and high BDNF group and 20% (24 of the 120 patients) in the low VCAM-1 and low BDNF group; the lowest prevalence of CKD (8.4%; 13 of 154 patients) was observed in the low VCAM-1 and high BDNF group (the *P* value for the trend was <0.001, **Figure 4**). In the univariate regression analyses, the highest OR of 10.846 (95% CI: 5.662–20.777, *P* < 0.001) for CKD was observed in the high VCAM-1 and low BDNF group, and it was followed by 5.840 (95% CI: 2.956–11.537, *P* < 0.001) in the high VCAM-1 and high BDNF group, and 2.712 (95% CI: 1.316–5.588, *P* = 0.007) in the low VCAM-1 and low BDNF group, compared with that in the low VCAM-1 and high BDNF group (**Table 4**). The risks of CKD were significantly associated with a decrease in serum BDNF levels and an increase in serum VCAM-1 levels compared to the first quartile groups.

**Table 4:** Odds ratios (95% CI) for chronic kidney disease in the univariate regression analyses.

	Odds ratio	95% CI	*P*
Study subgroups			<0.001
low VCAM-1 / high BDNF (n = 154)	1.000		
low VCAM-1 / low BDNF (n = 120)	2.712	(1.316, 5.588)	0.007
high VCAM-1 / high BDNF (n = 120)	5.840	(2.956, 11.537)	<0.001
high VCAM-1 / low BDNF (n = 154)	10.846	(5.662, 20.777)	<0.001
BDNF			<0.001
Quartile 1 (< 19.95 ng/mL)	1.000		
Quartile 2 (19.95-24.87 ng/mL)	0.481	(0.292, 0.794)	0.004
Quartile 3 (24.88-29.63 ng/mL)	0.297	(0.173, 0.509)	<0.001
Quartile 4 (≥ 29.64 ng/mL)	0.311	(0.182, 0.530)	<0.001
VCAM-1			<0.001
Quartile 1 (< 587 ng/mL)	1.000		
Quartile 2 (587-749 ng/mL)	2.683	(1.268, 5.678)	0.010
Quartile 3 (750-945 ng/mL)	5.790	(2.844, 11.788)	<0.001
Quartile 4 (≥ 946 ng/mL)	13.065	(6.477, 26.355)	<0.001

BDNF, brain-derived neurotrophic factor; CI, confidence interval; VCAM-1, vascular cell adhesion molecule-1.

To determine the independent association between CKD and these four subgroups, we selected confounding factors that were significantly associated with both CKD in **Table 1** and the BDNF or VCAM-1 levels in **Table 2**. Therefore, we included the variables, namely age, sex, total cholesterol, LDL cholesterol, HDL cholesterol, triglycerides, UACR, use of antihypertensive drugs, use of angiotensin-converting enzyme inhibitors or angiotensin II receptor blockers, use of calcium channel blockers, use of diuretics, use of insulin therapy, and use of metformin in the multivariate logistic regression analysis (**Table 5**). The highest OR of 3.885 (95% CI: 1.766–8.547, *P* < 0.001) for CKD was observed in the high VCAM-1 and low BDNF group, and it was followed by 3.099 (95% CI: 1.373–6.992, *P* = 0.006) in the high VCAM-1 and high BDNF group compared with that in the low VCAM-1 and high BDNF group. Although the OR for CKD was 1.634 (95% CI: 0.688–3.884) in the low VCAM-1 and low BDNF group, the difference was not statistically significant compared with the low VCAM-1 and high BDNF group (*P* = 0.266).

**Table 5:** Odds ratios (95% CI) for chronic kidney disease based on the associated risk factors in the multivariate regression analysis.

	Odds ratio	95% CI	*P*
Study subgroups			0.003
low VCAM-1 / high BDNF (n = 154)	1.000		
low VCAM-1 / low BDNF (n = 120)	1.634	(0.688, 3.884)	0.266
high VCAM-1 / high BDNF (n = 120)	3.099	(1.373, 6.992)	0.006
high VCAM-1 / low BDNF (n = 154)	3.885	(1.766, 8.547)	<0.001
Age			<0.001
Quartile 1 (< 56.3 years)	1.000		
Quartile 2 (56.3-63.0 years)	3.209	(1.287, 7.998)	0.012
Quartile 3 (63.1-69.4 years)	5.251	(2.193, 12.578)	<0.001
Quartile 4 (≥ 69.5 years)	25.243	(10.378, 61.398)	<0.001
Total cholesterol			0.819
Quartile 1 (< 3.5 mmol/L)	1.000		
Quartile 2 (3.5-4.0 mmol/L)	0.986	(0.473, 2.054)	0.970
Quartile 3 (4.1-4.5 mmol/L)	1.214	(0.506, 2.916)	0.664
Quartile 4 (≥ 4.6 mmol/L)	0.810	(0.274, 2.399)	0.704
LDL cholesterol			0.030
Quartile 1 (< 1.8 mmol/L)	1.000		
Quartile 2 (1.8-2.1 mmol/L)	1.152	(0.556, 2.384)	0.704
Quartile 3 (2.2-2.6 mmol/L)	0.410	(0.175, 0.960)	0.040
Quartile 4 (≥ 2.7 mmol/L)	0.449	(0.161, 1.254)	0.127
Triglycerides			0.064
Quartile 1 (< 0.88 mmol/L)	1.000		
Quartile 2 (0.88-1.34 mmol/L)	1.895	(0.898, 3.999)	0.093
Quartile 3 (1.35-2.02 mmol/L)	2.131	(0.994, 4.570)	0.052
Quartile 4 (≥ 2.03 mmol/L)	3.073	(1.338, 7.062)	0.008
UACR			0.001
Normal (< 30 mg/g)	1.000		
Moderate increased (30-299 mg/g)	1.949	(1.068, 3.555)	0.030
Severely increased (≥ 300 mg/g)	3.449	(1.743, 6.824)	<0.001
Male	2.344	(1.357, 4.050)	0.002
Low HDL cholesterol*	1.443	(0.818, 2.544)	0.205
Use of antihypertensive drugs	1.885	(0.651, 5.460)	0.243
Use of ACE inhibitor or ARB	0.781	(0.284, 2.147)	0.632
Use of calcium channel blocker	1.172	(0.521, 2.635)	0.702
Use of diuretics	1.955	(0.739, 5.173)	0.177
Use of insulin therapy	0.898	(0.529, 1.526)	0.692
Use of metformin	0.372	(0.216, 0.643)	<0.001

ACE, angiotensin-converting enzyme; ARB, angiotensin II receptor blocker; BDNF, brain-derived neurotrophic factor; BP, blood pressure; CI, confidence interval; HDL, high-density lipoprotein; LDL, low-density lipoprotein; UACR, urinary albumin-to-creatinine ratio; VCAM-1, vascular cell adhesion molecule-1.

* Low HDL cholesterol means an HDL level <1.0 mmol/L (40 mg/dL) in men or <1.3 mmol/L (50 mg/dL) in women.

## 4 Discussion

In the present study, we found that CKD in patients with type 2 DM was associated with low BDNF and high VCAM-1 levels in serum. Moreover, serum VCAM-1 levels were inversely correlated with BDNF levels, and high VCAM-1 levels had a synergistic effect with low BDNF levels on CKD in patients with type 2 DM. Hyperglycemia may downregulate BDNF expression in proximal tubular cells and enhance fibrosis in renal tissue (22, 23). Similar to the dendritic spines of neuronal cells, podocyte processes express tropomyosin-related kinase B receptor (TrkB), a specific BDNF receptor, and exhibit TrkB-dependent trophic activity by enhancing the phosphorylation of cofilin, a family of actin-binding proteins essential for maintaining normal podocyte architecture and facilitating structural changes in actin during the initiation and recovery phases of podocyte injury (24, 25). Podocyte injury following chemical damage can be repaired by administrating BDNF in the *in vitro* and animal models (26).

Circulating VCAM-1 levels are inversely correlated with eGFR and positively correlated with UACR in patients with type 2 DM (27). VCAM-1 functions as an adhesion molecule for leukocytes, facilitating the interaction of tubular proteins with mononuclear cells to promote interstitial fibrosis and subsequent CKD under diabetic conditions (28). Li et al. (29) also reported that high circulating VCAM-1 levels were associated with poor recovery of renal function after acute renal failure in patients with sepsis.

In addition to BDNF protecting against CKD and VCAM-1 promoting CKD development, Su et al. (30) reported that cultured human umbilical vein endothelial cells (HUVECs) exposed to uremic toxins in serum of patients with CKD inhibited the BDNF expression, and subsequently induced VCAM-1 expression and endothelial dysfunction in the *in vitro* study. This mechanism also explains the increased risk of CAD in patients with low serum BDNF and high serum VCAM-1 levels (17). In the present study, although the correlation between BDNF and VCAM-1 was only significant in the non-CKD group, the correlation coefficient in the CKD group, which had a relatively small sample size, was similar to that in the non-CKD group. The strength of the present study is that we showed a synergistic effect of high VCAM-1 and low BDNF levels on CKD in patients with type 2 DM.

Decreased circulating BDNF levels are associated with an increased risk of CAD, which is also associated with CKD (31). Furthermore, low BDNF levels can also be predictive of cardiovascular events and mortality (32–34). Similarly, excessive VCAM-1 expression in the arterial tissue has been observed in patients with advanced CKD, and serum VCAM-1 levels are predictive of cardiovascular events (35). Changes in BDNF levels are linked to the endothelial dysfunction, a crucial early step in atherosclerotic development (36). Atherosclerotic cardiovascular disease can induce inflammation and kidney injury (37, 38). CAD has been thought as an independent predictor of end-stage renal disease in patients with type 2 DM (39). Therefore, BDNF probably plays a protective role in inflammatory-vascular interactions involved not only in cognitive dysfunction but also in chronic cardiovascular and kidney disorders (17, 40, 41).

In the present study, old age and male sex were independent risk factors for CKD. In line with our findings, an increased prevalence of CKD was observed in old age and male population based on the National Health Insurance Research Database (NHIRD) (42, 43). In addition to hyperglycemia, hypertriglyceridemia was also reported as an important risk factor for CKD in patients with type 2 DM (44, 45). We found different patterns of medication use between patients with and without CKD in the present study. According to data from the Joint Asia Diabetes Evaluation Program, a higher proportion of patients used antihypertensive drugs and a lower proportion of patients used metformin were observed in the CKD group than that in the non-CKD group (46). Nonetheless, a synergistic effect of low BDNF and high VCAM-1 levels was observed after adjusting for these potential confounders.

Our study had some limitations. First, the cross-sectional design of the present study restricts the ability to establish causal correlations. Second, we recruited participants with poor glucose control, defined as having at least one HbA1c measurement exceeding 9.0% within the previous year. However, more than half (51.5%) of the enrolled patients had HbA1c levels <8.5% when blood samples were collected. Third, we did not directly explore the molecular pathways connecting BDNF and VCAM-1 to the CKD process. Finally, all participants were enrolled from the same teaching hospital. Therefore, further large-scale studies with longitudinal follow-up are needed to investigate the effects of BDNF and VCAM-1 on CKD in patients with type 2 DM.

## 5 Conclusion

Low serum BDNF and high VCAM-1 levels were significantly associated with CKD in patients with type 2 DM. Furthermore, serum VCAM-1 levels inversely correlated with BDNF levels and had a synergistic effect with low BDNF levels on CKD. The molecular pathway connecting BDNF and VCAM-1 may be involved in the CKD in patients with type 2 DM.

## Data Availability

The raw data supporting the conclusions of this article will be made available by the authors, without undue reservation.
